# Lifestyle Behaviours Add to the Armoury of Treatment Options for Panic Disorder: An Evidence-Based Reasoning

**DOI:** 10.3390/ijerph120607017

**Published:** 2015-06-18

**Authors:** Rod Lambert

**Affiliations:** School of Health Sciences and Health Economics Consulting, Faculty of Medicine and Health Sciences, University of East Anglia, Norwich NR4 7TJ, UK; E-Mail: r.lambert@uea.ac.uk; Tel.: +44-786-859-3305

**Keywords:** occupational therapy, occupational science, lifestyle behaviour, panic disorder, evidence based reasoning, multi-level analysis

## Abstract

This article presents an evidence-based reasoning, focusing on evidence of an Occupational Therapy input to lifestyle behaviour influences on panic disorder that also provides potentially broader application across other mental health problems (MHP). The article begins from the premise that we are all different. It then follows through a sequence of questions, examining incrementally how MHPs are experienced and classified. It analyses the impact of individual sensitivity at different levels of analysis, from genetic and epigenetic individuality, through neurotransmitter and body system sensitivity. Examples are given demonstrating the evidence base behind the logical sequence of investigation. The paper considers the evidence of how everyday routine lifestyle behaviour impacts on occupational function at all levels, and how these behaviours link to individual sensitivity to influence the level of exposure required to elicit symptomatic responses. Occupational Therapists can help patients by adequately assessing individual sensitivity, and through promoting understanding and a sense of control over their own symptoms. It concludes that present clinical guidelines should be expanded to incorporate knowledge of individual sensitivities to environmental exposures and lifestyle behaviours at an early stage.

## 1. Introduction

In 2013, the World Economic Forum produced a report that challenged the global healthcare community to seriously consider ways of delivering sustainable healthcare [1]. This report set the target for the UK as being to:

*“Shift healthcare out of hospitals into communities, spurring innovation through greater competition in delivery, introducing more humanized care into healthcare, and investing in behavioural change and prevention to diminish demand”*

([1] p. 9)

In 2010, the UK Government introduced the White Paper, “Healthy Lives, Healthy People” [2], in which, the opening statement in the foreword is that

*“We have to be bold because so many of the lifestyle-driven health problems we see today are already at alarming levels. Britain is now the most obese nation in Europe. We have among the worst rates of sexually transmitted infections recorded, a relatively large population of problem drug users and rising levels of harm from alcohol. Smoking alone claims over 80,000 lives every year. Experts estimate that tackling poor mental health could reduce our overall disease burden by nearly a quarter. ... We need a new approach that empowers individuals to make healthy choices and gives communities the tools to address their own, particular needs”*

([2] p. 2).

A recent Health Technology Assessment review of the cost-effectiveness of Health Advisors [3] concluded that there is a need to develop theoretically sound interventions that map to different population health needs. These need to be evaluated with increasing rigour, using the early stages of the MRC framework [4,5]. However, for this strategy to be effective, we also need well informed practitioners, who understand and engage with the complexities involved in lifestyle behaviours, to enable them to give more than a passing thought to lifestyle influences on health and mental health.

The College of Occupational Therapists are aware of the essential role that the profession’s philosophy has on health and well-being. It has produced its strategy document “Recovering Ordinary Lives” [6], which concludes that its goal is to ensure that *“mental health services are designed and delivered in ways that meet the occupational needs of the people who use them, thus promoting good mental health, assisting recovery and preventing mental ill health”* ([6] p. 21).

This article will examine selected evidence of complex interactions between genetic and epigenetic, neurotransmitter, body system, cognitive, environmental and lifestyle behaviour influences on panic disorder. It will present an evidence-based reasoning to show how lifestyle behaviours can add a substantial and cost-effective treatment option for the treatment of panic disorder (PD), and has the potential for much broader application across other mental health problems.

Literature from genetic variation and epigenetic interactions, neurotransmitter and body system sensitivity and sensitivity to lifestyle behaviours are related to a previous controlled trial, conducted and reported by the author [7,8,9,10,11]. Studies representing high levels of evidence are identified from the Cochrane Database, PubMed, AMED, ASSIA, CINAHL, EMBASE and PsychInfo. Keywords appropriate to each stage of the paper were used, but this overarching paper does not claim to be a systematic review or meta-analysis. The strongest evidence was extracted, discussed and synthesised at each stage. The main research was conducted between 2000 and 2003, while theoretical developments presented remain ongoing.

## 2. Background

The premise from which this article begins is that we are all different. The simplicity of this statement underlies a diverse range of inter-related mechanisms through which mental health problems (MHPs) can be viewed. However, it could be argued that this premise also underlies the reason why Occupational Science came into being. To examine the nature of human behaviour to understand why we make the choices we do, and how those choices then influence our nature, our interactions and our place in families, workplaces and communities. This article will examine some of the science behind just a few of these principles.

Current mental health treatment guidelines have polarised on medication at one pole, and psychological therapies at the other, either alone or in combination [12,13,14]. However, this article argues that an additional approach based on habitual lifestyle behaviours, can be effectively integrated with both forms of treatment. It is suggested that adaptation of habitual lifestyle behaviours can improve both theoretical and clinical understanding and broaden available treatment strategies for people with MHPs.

This argument is produced in the context of a developing debate on the complex nature of healthcare, whether conditions are chronic [15,16] or acute [17,18] in nature. The Medical Research Council framework for the development and evaluation of RCTs for complex interventions [5,19] places a high priority on investigating complex interactions in clinical contexts.

### An incremental Evidence-based Reasoning

The word “reasoning” is defined by the Oxford Dictionary of English [20] as “*the action of thinking about something in a logical, sensible way*”. The term “clinical reasoning” has been adopted widely in health care through which this form of thinking can be used to influence understanding and treatment in the clinical setting. In a 1986 Eleanor Clarke Slagel Lecture for Occupational Therapists, it was concluded that “the scientific, ethical and artistic dimensions of clinical reasoning are inextricably intertwined, and each strand is needed to strengthen the line of thought leading to understanding” [21]. Greater understanding of the principals and processes involved in clinical reasoning has subsequently developed, and the process is sometimes also classified as “Clinical Decision-making”. A recent characterisation of this, is that “*Clinical decision making is a contextual, continuous, and evolving process, where data are gathered, interpreted, and evaluated in order to select an evidence-based choice of action*” [22].

For the purposes of this article, it is therefore useful to begin by considering this philosophical position, as consideration is necessary of the complex interactions across not only different elements that influence PD, but also across different levels of analysis. To be able to present this argument in a coherent form, the application of reasoning according to the above definitions is necessary. This article will therefore apply reasoning to a number of propositions, and apply reason to the evidence with each proposition, in an incremental way. Only after evaluating the evidence and reaching a conclusion, will the next progression be made.

The author therefore, presents a series of incremental questions, each of which is examined for legitimacy before moving to the next. It is argued further that the questions are legitimate for general MHPs, but are focused in detail on PD. The series of questions to be examined are as follows, and are incremental in the sense that they begin from a relatively broad basis, and then focus on more specific elements which lead towards an examination of how and why habitual lifestyle behaviours may provide a cost-effective addition to the treatment armoury. The questions are:

- Are MHPs experienced as individualised sequences of symptoms, often with common and identifiable symptom profiles?
- Do genetic variation and epigenetic control mechanisms partially explain how/why system sensitivity may be altered?
- Is there evidence of altered sensitivity within neurotransmitter systems?
- Is there evidence of altered sensitivity within body systems?
- Does altered body system sensitivity lead to lower levels of environmental exposure being required to provoke symptomatic reactions?
- Are such reactions open to misinterpretation if the causes remain unrecognised?
- Can modification of lifestyle behaviours provide an effective intervention?

A specific clinical example of PD is used to illustrate and examine this argument, although similar evidence is available for other MHPs.

## 3. An Incremental Evidence-Based Reasoning

Each of the questions above will now be examined in detail.

### 3.1. Are MHPs Experienced as Individualised Sequences of Symptoms, Often with Common and Identifiable Symptom Patterns?

As stated, Panic Disorder will be used throughout as the exemplar, while it is argued the principles apply to other MHPs as well. Panic Disorder is defined within the Diagnostic and Statistical Manual of Mental Disorders, Revision IV Text Revision (DSM-IVTR) [23] as, “recurrent unexpected panic attacks” [23]. Panic attacks are defined as, a discrete period of intense fear or discomfort, in which four (or more) of the following symptoms developed abruptly and reached a peak within 10 min (American Psychiatric Association [23]):

- Palpitations, pounding heart, or accelerated heart rate
- Sweating
- Trembling or shaking
- Sensations of shortness of breath or smothering
- Feeling of choking
- Chest pain or discomfort
- Nausea or abdominal distress
- Feeling dizzy, unsteady, lightheaded or faint
- Derealization (feelings of unreality) or depersonalization (being detached from oneself)
- Fear of losing control or going crazy
- Fear of dying
- Parastheias (numbness or tingling sensations)
- Chills or hot flushes

Even without other evidence, from these 13 potential symptoms, over 700 possible combinations of four symptoms result. This provides a basis for suggesting broad variation of clinical presentation within the classification framework for PD. However, the issues of diagnosis are further complicated by a range of other factors. A study of 630 patients with PD, identified 10 factors in its development. These were “panic symptoms, agoraphobia, claustrophobia, separation anxiety, fear of losing control, drug sensitivity and phobia, medical reassurance, rescue object, loss sensitivity and reassurance from family members” [24].

Additionally, demographic factors also influence the condition, such as gender [25,26], cultural diversity [27] and age of onset [28,29], along with hypotheses such as a false suffocation alarm [30], and a range of suggested clinical subtypes such as fearful *vs.* non-fearful [31], respiratory subtype [32], a hyperventilation subtype [33], personality subtypes, “high functioning, emotionally dysregulated, inhibited/avoidant, and somatising” [34].

It should also be considered that PD develops within a developmental and a social context, where childhood learning and experiences such as separation and the associated anxiety can predispose a child to later PD [35]. Environmental influences both within the family [36], and in more general working and social environments can also influence levels of anticipatory or reactive anxiety [37].

This shows a broad variation of factors influencing individual symptom presentation in PD, while remaining within the recognised classification. With such a broad range of possible influences and presentations, it appears logical to reason that an equally broad range of causal factors may be influential. If this is the case, then a more diverse range of treatment strategies is required through which to provide effective interventions. The need for a broader range of new approaches to treatment has been previously recognised [38]. We will now begin to examine a range of levels of potential influence, beginning with whether genetic variation and epigenetic control mechanisms can help to explain this diversity.

### 3.2. Do Genetic Variation and Epigenetic Control Mechanisms Partially Explain How/Why System Sensitivity May Be Altered?

Vastly increased knowledge about genetic factors in disease has developed from the Human Genome Project [39]. New techniques in molecular biology have identified that “the extent of structural genomic variation is much greater than was previously suspected … a variable number of copies of a particular DNA segment are referred to as copy number variants” [40]. It has been suggested that it is “not normal to be walking around with the perfect genome” and that “about 1000 copy number variations exist in each person” [41]. Dawkins suggested that “Darwinian selection can function only if there is a good supply of genetic variation to work upon” [42], and that this variation can either promote positive or deleterious mutation [43].

In discussing a “paradox of vanishing variation” [42], Dawkins suggests that variation in characteristics are influenced by an indefinitely large number of different genes—“Polygenes”—whose small effects all add up. This argument itself supports the existence of individual sensitivity caused by genetic variation across multiple genes. A common theme in genetic studies of PD is for a multifactorial genetic influence rather than that of a single gene [44] and that multiple body systems may be involved as a result [45]. Predisposition for Panic Attacks and phobic anxiety may also be influenced by genes that affect anxious temperament and conditioned fear [46], rather than specifically through genes that predispose for the condition itself.

However, epigenetic control mechanisms also act at the gene-environment interface [47]. These epigenetic mechanisms have been likened with “perception” of, and adaptation to, environmental conditions, whether at cellular, system or individual/community levels [48] thereby demonstrating a fractal quality of repeating patterns at different levels of analysis. It is also recognised that epigenetic control mechanisms can have long term effects on our perceptions and experience of health, and influence learning and memory formation as well as the development of illness processes like cancer [49]. These mechanisms are known to be affected by diverse influences such as maternal care, diet, and exposure to toxins [49]. Animal models continue to examine the relationship between epigenetic controls and cognitive and mental health issues such as anxiety and depression, including the potential role of Glial Cell Line-Derived Neurotrophic Factor (GDNF) in the development and maintenance of individual coping strategies [50]. These mechanisms have also been shown to influence cognitive function through lifestyle behaviours such as exercise [51]. There is some initial evidence that patients with PD demonstrate alterations in sympathetic nervous system function which is controlled by epigenetic mechanisms involving phenylethanolamine N-methyltransferase (PNMT) [52].

Therefore, genetic variation along with variations in internal and external environmental conditions to which epigenetic control mechanisms respond, are common. These variations influence individual characteristic sensitivities, coping strategies and provides a partial explanation for the wide variation in presentation of MHPs, including PD. From this position, it is reasonable to ask how these variations may be expressed through individual sensitivity. A reasonable starting point for the next question therefore is at the level of neurotransmitter systems.

### 3.3. Is There Evidence of Altered Sensitivity within Neurotransmitter Systems?

Variable gene expression includes the synthesis and function of neurotransmitters [53]. Such gene expression for neurotransmitter function is under the control of epigenetic mechanisms that are responsive to environmental conditions [54]. A number of neurotransmitter/neuroreceptor systems have been implicated in PD, including acetylcholine [55], gamma aminobutyric acid (GABA) [56], dopamine [57], epinephrine [52], norepinephrine [58], serotonin [59] and the neuropeptide cholecystokinin [60]. If altered sensitivity in neurotransmitter systems is observed in patients with PD, this may indicate a mechanism through which PD itself may be expressed. For the purpose of illustration, we will examine the evidence related to epinephrine and serotonin, although similar results are obtained from the other neurotransmitter and neuroreceptor systems.

#### 3.3.1. Epinephrine

The catecholamines (dopamine, norepinephrine (NE) and epinephrine (EPI)) are all on the same synthetic pathway. All catecholamines are synthesised from the amino acid tyrosine, found in the diet and also from hydroxylation of phenylalanine, in the liver [61]. Tyrosine is converted first to dopamine then to NE and finally to EPI. Once released into the circulation, NE and EPI bind to the same adrenergic receptors [62].

EPI has been the subject of a greater number of RCTs than both dopamine and NE, and therefore the quality of evidence is potentially higher. Three reports from RCTs have used EPI infusion to provoke a panic reaction in over 60% of PD patients, compared with 5.6% with placebo [63,64,65].

In a placebo controlled RCT of Doxapram (a respiratory stimulant) with 16 PD patients and 16 normal controls [66], PD patients compared with controls were observed to have increased base levels of EPI, Adrenocorticotrophic Hormone (ACTH) and cortisol. In PD patients, a brief and pronounced EPI response was observed (within 2 to 5 min). Doxapram infusion was not associated with increased NE, Growth Hormone (GH), ACTH or cortisol reactions. A lower EPI response was observed in patients given a cognitive intervention than in patients provided with a standard intervention. The very brief EPI response period may plausibly have been missed by other studies. For this type of induced panic, or possibly for respiratory types of panic more generally, the cognitive intervention was able to attenuate the physiological and psychological reactions.

However, a RCT using EPI to provoke a panic reaction, with patients randomly allocated to groups with/without extensive pre-test information, and with/without perceived control over infusion, a fear of anxiety symptoms was not observed to predict panic reactions [63,65]. The authors concluded in their earlier RCT that in EPI-induced panic, cognitive factors may not be as important as physiological responses [63]. Levels of information provided and perceived control were not associated with panic reactions. The evidence indicates that more PD patients than controls are sensitive to the physiological effects of EPI, and that the brief pronounced reaction to EPI may play an important role in panic reactions. This mechanism has been used to investigate increased catecholamine levels which normalise after treatment with Paroxetine [67].

#### 3.3.2. Serotonin (5-HT)

Serotonin is produced from the precursor essential amino acid Tryptophan, in both the brain and the adrenal gland. Serotonin influences vascular tone [68], food intake, mood, motor output, learning, sleep, circadian pattern, aggression, sexual behaviour [69,70] and pain [71].

Serotonin is the most widely researched and reported neurotransmitter in PD, with selective serotonin reuptake inhibitors (SSRIs) currently promoted in clinical guidelines as the drug of choice [12,13,14]. However, a meta-analysis of effect-size from 12 placebo-controlled medication trials for PD [72] reported similar effect sizes for SSRIs and older antidepressants such as imipramine, and that larger studies were associated with a smaller effect size. Combined SSRI and CBT has been reported as superior in the number of post-treatment panic free patients, to either treatment provided singly [73].

There appears to be a link between serotonin and NE [74], cholecystokinin and GABA [75]. There is an acknowledged complex range of interactions involving plasma, cerebrospinal fluid (CSF) and platelet levels of serotonin and challenge agents such as cholecystokinin-4 (CCK-4), carbon dioxide and tryptophan depletion [76]. This includes alterations in cholecystokinin receptor sensitivity [77]. Lowering of brain serotonin has been shown under double blind conditions to increase panic reactions in PD patients [78], and that this may be gender specific [79]. The severity of PD has been associated with the 5HT2 receptor gene [80]. However, another report [81] suggests that dys-regulation of the 5-HT1A receptor may be implicated in alterations of the serotonin system. This may predispose individuals to mental illness, including PD, through its action in hippocampal, cortical, and hypothalamic regions involved in mood, emotion and stress response.

Serotonin represents an important factor in the predisposing vulnerability for PD, and in its interaction with a range of other neurotransmitter systems [75,82]. There is increasing evidence that patients with PD have altered sensitivity in their serotonin system at both neurotransmitter and receptor levels [25].

In summary, evidence supports genetically and epigenetically influenced variation in the sensitivity of a number of major neurotransmitter systems in patients with PD. Each of these neurotransmitter systems requires precursor essential amino acids from dietary intake. Interaction between these systems promotes further complex variability. Such variability may influence the symptom profile, severity and sub-typing of PD. However, it leads to the question of whether genetic and epigenetic variability, expressed through altered neurotransmitter sensitivity, continues to be expressed through alterations in the sensitivity of higher level body system function?

### 3.4. Is There Evidence of Altered Sensitivity within Body Systems?

At least 6 major body systems have been implicated in PD (Respiratory, Cardiac, Audiovestibular, Gastrointestinal, Musculo-skeletal and Immune systems [10]. The evidence in this section will be examined for the respiratory and immune systems, while similar evidence exists for the other systems mentioned. Each of these systems in turn, link to lifestyle behaviours that may influence function.

#### 3.4.1. Respiratory System

It has been suggested that the lifetime prevalence of panic attacks with a respiratory component is 6.77%, compared with panic attacks without a respiratory component of 3.14% (12-month figures are 2.26% and 1% respectively) [83]. Despite many calls for a respiratory subtype of PD [32,84,85], this recent work showed insufficient differences between respiratory and non-respiratory forms to warrant a specific sub-typing. However, there is considerable evidence to suggest that the respiratory system in patients with PD has an altered level of sensitivity compared with other patients and normal controls [32,86]. There is also some evidence of a differential treatment response between patients classified as respiratory subtype PD compared with those with Non-Respiratory Subtype PD [87]. This section examines three main elements of the respiratory system, CO_2_ sensitivity, sodium lactate and hyperventilation.

CO_2_ inhalation consistently provokes panic attacks in PD patients [88,89]. PD patients with increased respiratory sensitivity appear to have greater panic reactions to CO_2_ inhalation, than PD patients without respiratory sensitivity. This is also true of sodium lactate infusion (See Table 1), with a mean of 77.7% of patients experiencing a panic reaction to sodium lactate infusion compared with 17.4% of controls.

**Table 1:** Studies of lactate-induced panic attacks in PD patients and controls.

Author (Year) [reference]	Study Type	PD Patients	Controls	% Lactate Induced Panic Attacks	
				Patients	Controls
Pitts & McClure (1967) [90]	RCT	14	10	93	20
Gorman *et al.* (1984) [91]	Cohort	12		67	
Fyer *et al.* (1985) [92]	RCT	13		58	
Liebowitz *et al.* (1985) [93]	RCT	43	20	72	0
Ehlers *et al.* (1986) [94]	RCT	10	10	90	60
Balon *et al.* (1988) [95]	RCT	86	45	85	22
Gaffney *et al.* (1988) [96]	Cohort	10	10	80	0
Gorman *et al.* (1988) [97]	Cohort	31	25	58	20
Aronson *et al.* (1989) [98]	Cohort	9	9	100	0
Balon *et al.* (1989) [99]	Cohort		45		22.2
den Boer *et al.* (1989) [100]	RCT	15	15	73	0
Russel *et al.* (1991) [101]	Cohort	11		100	
Goetz *et al.* (1996) [102]	Cohort	202		59	
Binkley & Kutcher (1997) [103]	RCT	5		100	
Coplan *et al.* (1998) [104]	RCT	170	44	59	23
Kellner *et al.* (1998) [105]	RCT	10	10	70	20
Strohle *et al.* (1998) [106]	RCT	10		80	
Strohle (2000) [107]	RCT	30	23	76.6	21.7
Total Mean		40.05882	22.16667	77.68235	17.40833
Lowest		5	9	58	0
Highest		202	45	100	60

Explanations of the underlying mechanisms for this vary between an altered function of neural circuitry in PD patients [108], a “multiple factors theory of central chemosensitive signalling” [109] with implications for metabolic factor involvement, and brain lactate responses to visual stimulation [110].

However, CO_2_ sensitivity should not be viewed in isolation. Studies using CO_2_ as a provocation agent, have observed that physiological states and environmental elements such as premenstrual distress [111] and perceived threats [112], can increase the panic response, while other components, such as exercise [89], can mediate the panicogenic effects of CO_2_ inhalation. It has also been posited that abnormal regulation of the respiratory system may modulate reactivity to lifestyle-related activity such as smoking and exercise levels [113].

Hyperventilation has been posited as indicative of hypersensitivity in a central “alarm” system [114] or as a false suffocation alarm [115,116]. A RCT of 11 PD patients compared with 5 first-degree relatives of PD patients and three controls reported higher sensitivity to hyperventilation in PD patients [117]. The underlying causal mechanism has not been defined, although possible routes identified include atrial natiuretic hormone (ANH) [118]; sympathetic stimulation [64]; greater basilar artery response [119]; the vascular response to hypocapnia [120,121]; alterations in breathing (not specific to CO_2_) [122] and cholinergic function [55,121]. Hyperventilation has been suggested as a further subtype of PD [85,86].

The notion of a false suffocation alarm has been supported by results from a family study of 104 PD probands and 247 first-degree relatives [123]. Relatives of PD probands with smothering sensations had an almost three-times-higher risk for panic, and almost six-times-higher risk of panic with smothering sensations compared with relatives of PD probands without smothering sensations. As in other hypotheses, the false suffocation alarm should not be considered in isolation, and a “framework connecting PD data to endogenous opiodergic dysfunction, separation anxiety, and respiratory vulnerabilities” [116] has been suggested as amplifying the false suffocation alarm theory.

This evidence indicates that altered sensitivity in the respiratory system is implicated in PD, and can manifest through CO_2_ sensitivity, hyperventilation, a hypersensitive fear network [32] and/or increased sensitivity to sodium lactate infusion [110]. In addition, respiratory variability may also provide a diagnostic marker for PD [124]. There is clearly an interaction between the respiratory system and other body systems, and behavioural and situational components that can both increase vulnerability to panic responses, and provide protective properties.

#### 3.4.2. The Relationship between PD and the Immune System

The human stress response is comprised of fluid homeostasis, glucose metabolism and immune function. The function of the immune system is to destroy extrinsic and potentially harmful invasions, while leaving the intrinsic elements intact [125,126]. There are two main ways in which it functions:

1 By destroying potentially toxic micro-organisms (antigens).
2 By creating an increased reaction to the presence of a previous invading agent.

There is increasingly strong genetic evidence to suggest a direct link between altered immune function and PD [127,128]. However, the strongest clinical evidence of altered immune function in PD patients is provided in a systematic review of 14 studies carried out between 1991 and 2002 [129]. This review provided moderate evidence that PD patients have increased numbers, but not increased activity in B-cells (specialised lymphocytes), indicating a possibility that these cells have become exhausted possibly due to earlier hyperactivity, but the authors recommended further study. There is some evidence to suggest that there is an inter-relationship between panic disorder and the T-cell population [130]. There is also a suggestion of bidirectional communication between the immune system and the Hypothalamic-Pituitary-Adrenal (HPA) axis in patients with PD, reflecting altered function in the immune system [131].

A randomised study of 343 offspring of PD patients, compared with non-panic psychiatric patients and normal controls [132], observed an increased risk of atopic disorders in both PD (Odds Ratio 2.56; 95% CI 1.27–5.16; *p* = 0.009) and separation anxiety disorder (Odds Ratio 2.71; 95% CI 1.22–6.03; *p* = 0.015). Childhood separation anxiety is a known precursor for adult panic disorder, with associations also with higher CO_2_ sensitivity [133].

The outcome of such altered immune function may be indicated through an increased incidence of allergic conditions, and increased susceptibility to medical conditions such as cancer. In a re-evaluation of the Epidemiological Catchment Area (ECA) data from both 1980–1981 and 1994–1996, moderate levels of evidence were reported that respiratory forms of panic attack were predictive of a higher risk of breast cancer and myocardial infarct [134]. In addition, case study reports and case-note reviews suggests that PD may predate the diagnosis of cancer in between 20% and 50% of cancer patients [135,136,137,138]. In a sample of 400 adult cancer patients, the prevalence of panic disorder was higher in cancer patients than in the general population [139].

These findings provide broad evidence that altered physiological sensitivity in patients with PD influences immune function. It is reasonable to suggest at this point that lifestyle interventions with PD patients may therefore identify potential immune system stressors, and suggest ways to reduce exposure to them, and to strengthen immune function through promoting a healthier lifestyle.

In summary, homeostatic and stress response mechanisms act to maintain system function within threshold limits. Altered sensitivity at different levels within each of these systems is seen to increase the likelihood of perceived altered bodily sensations at lower levels of provocation than in conditions of “normal” sensitivity. At least six body systems, and a range of neurotransmitter systems have been shown to have altered sensitivity in PD patients.

The ability of PD subtypes to mimic other somatic conditions continues to be recognised [140]. The clinical utility of identifying specific subtypes remains under investigation [141,142]. While not exhaustive, the evidence presented supports that body system sensitivity at one or more levels of analysis, is altered in a proportion of patients with PD. The potential impact of such body system sensitivity may be to lower the provocation required from exposure to a range of factors, to elicit symptomatic reactions, and this now needs to be examined.

### 3.5. Does Altered Body System Sensitivity Lead to Lower Levels of Environmental Exposure Being Required to Provoke Symptomatic Reactions?

This question will be examined through discussion of the same two previously discussed body systems (Respiratory and Immune systems), although the principles again can be applied across all six body systems mentioned. Through this, it is possible to examine whether there is evidence to support symptomatic reaction to lower levels of provocation.

#### 3.5.1. Respiratory System Reactivity

Normal respiration is dependent upon balance being maintained between inspired oxygen (O_2_), expired carbon dioxide (CO_2_) and blood Ph (normally 7.4 ± 0.05). Two main factors influence this:

1. Respiration rate, maintaining O_2_/CO_2_ balance, thereby maintaining blood Ph at close to normal.
2. Change in metabolic rate, from whatever cause.

When the normal O_2_/CO_2_ balance is disturbed, a homeostatic negative feedback response tries to return the system to within its normal limits. Hyperventilation excretes CO_2_ faster than it is produced; leading to decreased arterial CO_2_ and *respiratory alkalosis* (pH rises above 7.45). Hypoventilation reduces elimination of CO_2_, leading to *respiratory acidosis* (pH falls below 7.35) [126]. Similarly, changes in metabolism can lead to *metabolic acidosis* or *metabolic alkalosis* [143].

A cross-sectional study of 35 PD patients [144] produced higher sodium lactate levels during intensive exercise than during sodium lactate infusion. Although less fit than the general population, only one patient (4%) experienced a panic attack during testing, compared with a general level of around 67% with sodium lactate infusion. Increased sodium lactate initiated by exercise or activity, provokes metabolic acidosis, while infusion of sodium lactate provokes systemic alkalosis [145]. It has been shown that adaptive responses to acute respiratory alkalosis brought about through hyperventilation, is exaggerated in panic disorder patients [146]. Conversely however, Martinsen *et al.* [144] suggested that PD patients might safely undertake submaximal and even maximal exercise without increased risk of panic reactions as the resulting acidosis does not appear to cause a panic response. Increased exercise can also improve respiratory function and potentially reduce respiratory-related panic reactions [89], and therefore can safely be used in treatment [147]. In a study comparing ten PD patients with ten normal controls [148], patients were observed to be less fit, and to reach physiological markers earlier when exercising than normal controls. There were however, no differences in the psychological variables.

#### 3.5.2. Immune System Reactivity

The skin acts as the first line of defence against external infection, while the body linings (such as in the respiratory and digestive systems) provide defence against inhaled or ingested antigens or allergens [149]. Disease and poor nutrition form a downward spiral of malnutrition and weakened immunity. Consistently strong evidence of a negative effect on immune function has been reported for smoking (but reversible when smoking ceases) [150], and excess alcohol use [151,152,153].

There has also been consistently strong evidence of a direct relationship between body weight, sickness symptoms and associated immune responses of [154]. While the negative impacts of smoking, alcohol and increased weight have been shown to be reversible, it has also been shown that positive behaviours such as routine exercise directly impacts positively on immune function and has a largely anti-inflammatory effect [155]. Lifestyle behaviours therefore have a strong relationship with immune function, which can both increase health risks, and also provide important and strong protective influences.

In animal models [156], an increased sensitivity in immune responses has been observed in anxiety prone mice compared with wild mice when challenged with Yohimbine and caffeine. It was concluded that altered sensitivity in the “anxiety- and panic-related circuitries, such as the thalamus, hypothalamus, amygdala and cingulate cortex” [156], may have been influential. This suggests that increased sensitivity to the anxiogenic effects of caffeine in panic disorder patients may influence the level of provocation required to influence the panic-related circuitry in these brain areas in human subjects. A recent systematic review demonstrated the positive association between caffeine and an anxiogenic reaction in PD patients compared with controls [157].

In summary, the evidence presented supports that altered physiological sensitivity in the two systems reported, and in at least six body systems overall, leads to lower levels of environmental provocation being required to elicit symptomatic reactions in patients with panic disorder. However, the next part of the investigation needs to explore whether such symptomatic reactions are obvious to those experiencing them, or whether they are frequently misinterpreted.

### 3.6. Are Such Reactions Open to Misinterpretation If the Causes Remain Unrecognised?

The cognitive approach to PD [158] is that panicogenic agents (such as lactate, isoproterenol, caffeine, hyperventilation and carbon dioxide) can provoke a panic reaction only if an associated catastrophic misinterpretation of the bodily sensations provoked by the agent is also present. Catastrophic misinterpretation leads to further apprehension and bodily sensations through a positive feedback loop [159]. A systematic review of the efficacy of CBT [160] concluded that the psychological process leading to a misinterpretation of bodily sensation is unclear, and that further research is required to determine whether the misinterpretation is prompted by a physiological symptom, or its cognitive perception.

It has been proposed [9] that the interaction between altered body system sensitivity, and adverse habitual lifestyle behaviours in patients with PD may provoke cognitive awareness of altered physiological bodily sensations at an earlier stage than for other groups. If this awareness of altered bodily sensations is then misinterpreted in the way that Clarke [158] suggested, then the spiral into a panic reaction is a logical conclusion. However, this differs from the purely cognitive model in important ways:

- Epigenetic control mechanisms are reactive to external environmental conditions, and therefore influence sensitivity to physiological sensations. Lifestyle behaviours such as diet, fluid intake, exercise and habitual lifestyle drug use (nicotine, caffeine and alcohol), directly influence bodily environmental conditions, and therefore also the function and cognitive appraisal of body systems.
- If the trigger for misinterpretation is from misunderstanding an altered physiological reaction, in addition to a purely psychological response, then the importance of understanding the cause of the panic reaction increases.
- If a rational explanation of the physiological nature of the sensation can be provided, and tested by the patient, through addressing habitual behaviours that may be influencing the physiological environment, then fear of the symptoms and sensations can be reduced.
- If patients can be assisted in recognising lifestyle behaviours to which they have an altered sensitivity, then the impact of change in habitual lifestyle behaviours can improve health status across a broader range of indicators. Control over symptoms can pass back to the patient through positive changes in environmental conditions.

In summary, if the causes of altered bodily sensations remain unknown, then misinterpretation is common, and often provokes anxiety/panic reactions. It is important to determine therefore, if rational explanations for experienced physiological symptoms, along with positive strategies to actively regain control over them, may provide an effective intervention?

### 3.7. Can Modification of Lifestyle Behaviours Provide an Effective Intervention?

A randomised controlled trial of an occupational therapy-led lifestyle approach to treating panic disorder in primary care compared with routine GP care [9] was conducted with 31 lifestyle arm and 36 GP care arm patients. Lifestyle behaviours investigated were dietary pattern, fluid intake, habitual lifestyle drug use and exercise. Results showed a significant reduction in Beck Anxiety Inventory (BAI) scores at the end of treatment (*p* < 0.001), although these were non-significant at a 10-month follow-up (*p* = 0.167). In two-thirds of lifestyle arm patients, further improvement was observed at the 10-month follow-up, suggesting that there is some carry-over effect from a 16-week period of monitored and supported lifestyle change in a proportion of patients. In comparison with other treatments reported using the BAI as an outcome measure, results were very similar to that achieved by full CBT, and superior to that achieved by medication, computerised CBT or routine care [9]. A cost-effectiveness analysis of this approach [7], considered there to be an 86% chance for it to provide value-for-money over 10 months when compared with routine GP care.

In an epidemiological study of 42,448 people in the Swedish population [161], physical activity, being underweight and risk consumption of alcohol were identified as independent factors in mental health symptoms. Although it has been reported that “persons reporting poor mental health were more likely to report unhealthy lifestyle behaviors” [162]. Successful clinical application of this lifestyle-based approach has not only been reported in panic disorder [7,9], but also for Obsessive Compulsive Disorder [163] and depression [164].

**Figure 1:**
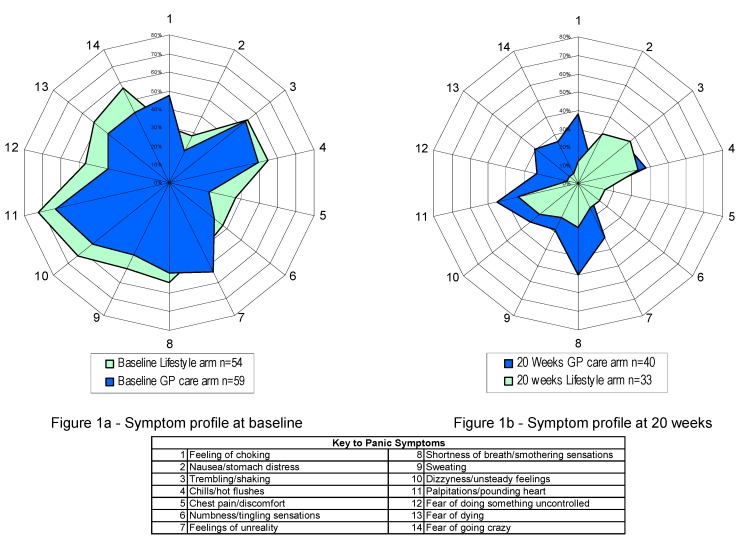
Symptom change at baseline and 20 weeks in GP care and lifestyle groups. Reproduced from Lambert *et al*. [8] with permission from Emerald Publishing.

In summary, the evidence presented indicates that physiological pre-disposition combined with poor lifestyle behaviours can impact on mental health problems. This is shown in evidence that although symptom profiles in PD patients were similar at baseline [8], between patients receiving a lifestyle intervention and routine GP care, there was a significant difference at the end of treatment (*p* = 0.008). The symptom profile in lifestyle arm patients reflected improved change in most symptoms compared with primary care, but the cognitive symptoms improved very markedly, indicating that rational explanations for symptoms improves their cognitive appraisal (See Figure 1).

If individual system sensitivity can be identified through lifestyle review, then this may provide a therapeutic opportunity to minimise exposure, or to improve fitness and function in these systems. In this way, perturbation of physiological processes can be modified, and increases the perception of individual control over previously misinterpreted symptoms. A range of practical lifestyle and occupational behaviours can then be established through which to reduce the physiological responses and thereby reduce anxiety and panic responses caused by a combination of altered physiological sensations and their misinterpretation.

## 4. Discussion

**Figure 2:**
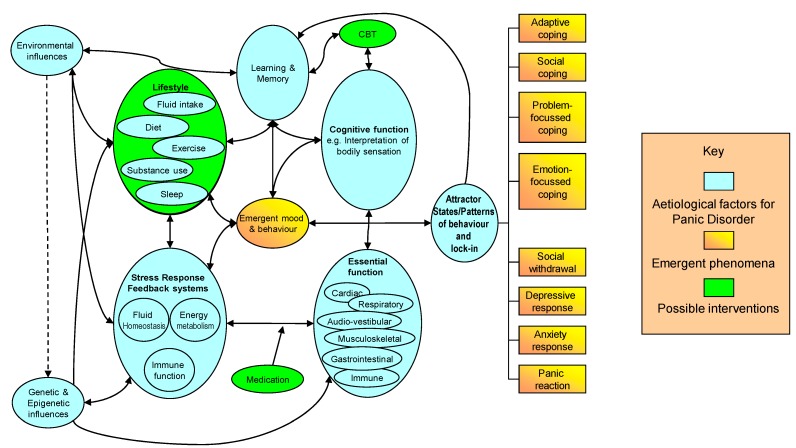
Complex interactions and Panic Disorder Emergent phenomena and Therapeutic implications. Reproduced and adapted from Lambert [165] with permission from Radcliffe Publishing.

Current clinical guidelines for treatment of mental health problems focus on medication and psychological therapies, either alone or in combination. This polarisation is challenged by the evidence of diverse and complex aetiology and presentation, including from genetic predisposition, epigenetic control mechanisms and altered physiological sensitivity in neurotransmitter and body systems. By viewing the interaction between physiological sensitivity and reactivity, cognitive appraisal and the influence of habitual lifestyle behaviour patterns, it becomes possible to identify increased opportunities for effective, early stage interventions. A model for this approach has been reported [165] and is reproduced in a modified form, and adapted here with permission (see Figure 2).

This model shows the many factors that influence mood and behaviour either directly or indirectly. This ranges from genetic/epigenetic and environmental influences, through impact of the tripartite physiological human stress response mechanisms (fluid homeostasis, energy metabolism and immune function) and the sensitivity and function of major body systems, to cognitive function including learning and memory. Habitual lifestyle behaviours also have an important role to play, acting both directly and indirectly on mood and behaviour. It should be remembered however, that mood and behaviour are not displayed randomly. Across cultures, it is possible to identify mood and behaviour that consistently show specific patterns and presentations, such as happiness or sadness. This is also true between individuals, as we all demonstrate moods and behaviours differently, dependent upon many factors from our past experiences, through to our current state of mood and health. While many of these patterns are adaptive, that is they enable us to cope with the pressures and challenges of everyday life, others have more negative connotations, leading to social implications, anxiety, depression and for the examples given, can include panic reactions. Like many moods and behaviours, the more often the pattern occurs, the more it becomes reinforced and likely to recur under circumstances that are actually or perceived to be similar.

From this perspective, it is possible to see rational points in the system at which the currently recommended medications and psychological therapies interact. However, habitual lifestyle behaviours already form an integral part of this system, and patients can be encouraged to review and change behaviours linked to identified individual sensitivity if a focused intervention is provided. It is proposed that this provides an effective additional and potentially early stage, intervention. It can help patients to identify environmental, interpersonal and social factors that may underlie heightened arousal or anxiety reactions. It can help patients to identify specific physiological sensitivities through which lifestyle changes can be promoted to help them regain control over some symptoms, thereby enhancing well-being and self-efficacy. This can also be cost-effective in reducing health care utilisation through medication use and also reduce some of the pressure on psychological services.

## 5. Conclusions

This article began from the proposition that we are all different. The article demonstrates an evidence based reasoning showing that individual differences are represented at all levels of system function and reactivity. Levels of system involvement are from genetic predisposition and variation, along with epigenetic control mechanisms, which are translated into variation in neurotransmitter and body system function. In turn, these sensitivities lead to individualised reactions to substances (such as caffeine and nicotine) and states (such as dehydration and allergic reactions). These sensitivities are partially genetically determined, and partially epigenetically controlled through either single episode or habitual exposure to lifestyle behaviours to which the individual is sensitive and reactive. Improving patient knowledge of sensitivities and their clinical impact can provide patients with coherent and evidence-based explanations and strategies through which they can regain control over mental health problems such as anxiety and panic, and provide constructive feedback into the control mechanisms. Careful examination of sensitivity and reactivity across a range of habitual lifestyle/occupational behaviours is more likely to lead to continued and persistent behavioural and physiological change, and through this, to symptom improvement. By improving knowledge and understanding of the underlying neuroscience behind everyday routine occupational behaviours, Occupational therapists can influence the way in which Mental Health Services are designed and delivered, and can meet the College of Occupational Therapists strategy aim to “meet the occupational needs of the people who use them, thus promoting good mental health, assisting recovery and preventing mental ill health” [6].

Present clinical guidelines should be expanded to incorporate knowledge of individual sensitivities to environmental exposures and lifestyle behaviours at an early stage. Further research is needed into how the proposed model fits with the evidence base for panic disorder and other mental health problems.
